# *Lactobacillus acidophilus* LA5 improves saturated fat-induced obesity mouse model through the enhanced intestinal *Akkermansia muciniphila*

**DOI:** 10.1038/s41598-021-85449-2

**Published:** 2021-03-18

**Authors:** Thunnicha Ondee, Krit Pongpirul, Peerapat Visitchanakun, Wilasinee Saisorn, Suthicha Kanacharoen, Lampet Wongsaroj, Chitrasak Kullapanich, Natharin Ngamwongsatit, Sarn Settachaimongkon, Naraporn Somboonna, Asada Leelahavanichkul

**Affiliations:** 1 Department of Preventive and Social Medicine, Faculty of Medicine, Chulalongkorn University, Bangkok, 10330 Thailand; 2 Department of International Health, Johns Hopkins Bloomberg School of Public Health, Baltimore, MD USA; 3 Bumrungrad International Hospital, Bangkok, 10110 Thailand; 4 Department of Microbiology, Faculty of Medicine, Chulalongkorn University, Bangkok, 10330 Thailand; 5 Department of Biology, Krieger School of Arts and Sciences, Johns Hopkins University, Baltimore, MD USA; 6 Department of Microbiology, Faculty of Science, Chulalongkorn University, Bangkok, 10330 Thailand; 7 Microbiome Research Unit for Probiotics in Food and Cosmetics, Chulalongkorn University, Bangkok, 10330 Thailand; 8 Department of Clinical Sciences and Public Health, Faculty of Veterinary Science, Mahidol University, Nakhon Pathom, 73170 Thailand; 9 Department of Food Technology, Faculty of Science, Chulalongkorn University, Bangkok, 10330 Thailand; 10 Emerging Processes for Food Functionality Design Research Unit, Chulalongkorn University, Bangkok, 10330 Thailand; 11 Translational Research in Inflammation and Immunology Research Unit (TRIRU), Department of Microbiology, Chulalongkorn University, Bangkok, 10330 Thailand

**Keywords:** Microbiome, Dyslipidaemias, Non-alcoholic fatty liver disease, Non-alcoholic steatohepatitis, Obesity

## Abstract

Obesity, a major healthcare problem worldwide, induces metabolic endotoxemia through the gut translocation of lipopolysaccharides (LPS), a major cell wall component of Gram-negative bacteria, causing a chronic inflammatory state. A combination of several probiotics including *Lactobacillus acidophilus* 5 (LA5), a potent lactic acid-producing bacterium, has previously been shown to attenuate obesity. However, data on the correlation between a single administration of LA5 versus microbiota alteration might be helpful for the probiotic adjustment. LA5 was administered daily together with a high-fat diet (HFD) for 8 weeks in mice. Furthermore, the condition media of LA5 was also tested in a hepatocyte cell-line (HepG2 cells). Accordingly, LA5 attenuated obesity in mice as demonstrated by weight reduction, regional fat accumulation, lipidemia, liver injury (liver weight, lipid compositions, and liver enzyme), gut permeability defect, endotoxemia, and serum cytokines. Unsurprisingly, LA5 improved these parameters and acidified fecal pH leads to the attenuation of fecal dysbiosis. The fecal microbiome analysis in obese mice with or without LA5 indicated; (i) decreased Bacteroidetes (Gram-negative anaerobes that predominate in non-healthy conditions), (ii) reduced total fecal Gram-negative bacterial burdens (the sources of gut LPS), (iii) enhanced Firmicutes (Gram-positive bacteria with potential benefits) and (iv) increased Verrucomycobia, especially *Akkermansia muciniphila*, a bacterium with the anti-obesity property. With LA5 administration, *A. muciniphila* in the colon were more than 2,000 folds higher than the regular diet mice as determined by 16S rRNA. Besides, LA5 produced anti-inflammatory molecules with a similar molecular weight to LPS that reduced cytokine production in LPS-activated HepG2 cells. In conclusion, LA5 attenuated obesity through (i) gut dysbiosis attenuation, partly through the promotion of *A. muciniphila* (probiotics with the difficulty in preparation processes), (ii) reduced endotoxemia, and (iii) possibly decreased liver injury by producing the anti-inflammatory molecules.

## Introduction

Obesity, a major healthcare problem worldwide^1^, is associated with diabetes, dyslipidemia, and cardiovascular disease that leads to several major complications in critically-ill patients^1^. Interestingly, obesity-induced chronic inflammation leads to atherosclerosis which is a key vascular complication in obesity^2^. Despite the prevalence of obesity-induced inflammation, the pathogenesis of this condition is still uncertain but is possibly a combination of several mechanisms including (i) immune responses toward adipocyte injury from hypertrophic adipocyte hypoxia and adipocyte apoptosis^3,4^, (ii) reduction of adiponectin together with leptin elevation, (iii) metabolic dysregulation and mitochondria dysfunction and (iv) gut permeability defect (gut-leakage) induced endotoxemia (metabolic endotoxemia)^5^. Among these, the interaction against endotoxins may lead to the most potent response because immune activation by the organism’s pathogen-associated molecular patterns (PAMPs) is naturally more severe than the response towards the host cell’s damage-associated molecular patterns (DAMPs)^6^. Endotoxin (lipopolysaccharide; LPS) has a molecular weight of 10–100 kDa and is found in the cell walls of Gram-negative bacteria, which are the most abundant organisms of gut microbiota^7^. Under normal conditions, molecules weighing over 600 Da are unable to pass through the intestinal tight junction barrier. However, obesity and a high-fat diet (HFD) cause gut dysbiosis^8^ (an alteration of organisms in the intestine^9^), enhancing gut-mucosal injury enough to allow the direct translocation of high molecular weight (MW) molecules, such as LPS, into the liver and circulatory system^10,11^. Indeed, non-alcoholic fatty liver disease (NAFLD), also known as obesity-induced steatohepatitis, is a key complication in obesity that is worsened by LPS^12^.

However, gut-leakage in obesity^10^ is attenuated by host-beneficial probiotics^13–15^, partly through the reduction of endotoxemia and systemic inflammation. Additionally, some probiotics produce high MW anti-inflammatory substances^16–20^. Thus, beneficial molecules secreted by probiotics may also be delivered alongside harmful PAMPs into the circulatory system during gut leakage. Although (i) *Lactobacillus* spp. attenuates gut dysbiosis in several animal models^20–25^ and (ii) *Lactobacillus acidophilus* (LA), in combination with other probiotics, attenuates hyperlipidemia, overweight and obesity-induced hepatitis (steatohepatitis)^12,26–29^, the effect of LA alone upon obesity and steatohepatitis is still unclear. As such, it is easier to determine the impact of microbiota alteration after the administration of probiotics in a single strain than a combination of several strains. The promotion of beneficial gut microorganisms in response to probiotics may be as important as the advantageous effect of probiotics themselves^30^. Fascinatingly, commercial probiotics can be administered to target the growth of difficult-to-culture bacteria in the gut. For instance, strictly anaerobic *Akkermansia muciniphila* can be promoted by commonly-available *Lactobacilli*^31^. Hence, *Lactobacillus acidophilus* LA5 (LA5) was used as a single strain probiotic in a mouse model with saturated fat induced-obesity and was also tested in the hepatocyte cell-line experiments.

## Materials and methods

### Animals and animal model

The animal care and use protocol was approved by the Institutional Animal Care and Use Committee of the Faculty of Medicine, Chulalongkorn University, Bangkok, Thailand (SST 025/2563) in compliance with US National Institutes of Health standards. This study was carried out in compliance with the ARRIVE guidelines. The 8-week-old male C57BL/6 mice were purchased from the National Laboratory Animal Center, Nakhorn Pathom, Thailand. Mice with a regular diet with standard laboratory chow containing fat (4.5% w/w) with energy content calculated at 3.04 kcal/g (Mouse Feed Food No.082, C.P. Company, Bangkok, Thailand) or high-fat diet (HFD) containing fat, mostly from lard (60% w/w), with energy content calculated at 8.64 kcal/g^32^ were orally administered *Lactobacillus acidophilus* LA5 (LA5) (Chr. Hansen, Hørsholm, Denmark) daily at 1 × 10^8^ colonies forming unit (CFU) in 0.5 ml phosphate buffer solution (PBS) or PBS alone for 2 months before sacrifice with cardiac puncture under isoflurane anesthesia. Livers were snap-frozen in liquid nitrogen and kept at −80 °C before use. Feces from all parts of the colon were combined and collected for microbiome analysis. Besides, fecal samples with the intestinal tissue, including the duodenum (distal to the pyloric sphincter), jejunum (central section of the small intestine), ileum (proximal to the cecum), and colon (distal to the cecum), from obese mice with or without LA5 were collected to measure *Lactobacillus* spp. and *Akkermansia* spp. content. Organs were weighed, homogenized, and centrifuged to separate the supernatant for bacterial DNA detection.

### Gut leakage measurement

Gut permeability was determined by fluorescein isothiocyanate dextran (FITC-dextran) assay, endotoxemia, and immunofluorescent detection of a tight junction protein (zonaoccludens-1; ZO-1) following previous publications^33–35^. As such, FITC-dextran, a nonabsorbable molecule with 4.4 kDa molecular mass (Sigma-Aldrich, St. Louis, MO, USA) at 12.5 mg per mice was orally administered at 3 h before the detection of FITC-dextran in serum by Fluorospectrometer (NanoDrop 3300; Thermo Fisher Scientific, Wilmington, DE, USA). Serum endotoxin (LPS) was measured by HEK-Blue LPS Detection (InvivoGen, San Diego, CA, USA) and the data were recorded as 0 when LPS values were less than 0.01 EU/ml because of the limited lower range of the standard curve. Also, the cecum was used as a representative of the intestine to determine gut tight junction. Accordingly, cecum in Cryogel (Leica Biosystems, Richmond, IL, USA) were cut into 5 μm-thick frozen sections, fixed in acetone, blocked by blocking buffer, stained with a fluorescent antibody against ZO-1 and a green secondary antibody (Alexa Fluor 488) (Life Technologies, Carlsbad, CA, USA) before visualization and scoring by ZEISS LSM 800 (Carl Zeiss, Germany).

### Analysis of mouse samples from blood, organs, and feces

After fasting for 12 h after free access to drinking water, lipid profiles were measured by the quantification assay for triglyceride (TG), total cholesterol (Sigma-Aldrich), low- and high- density lipoprotein cholesterol (LDL and HDL) (Crystal Chem Inc., Downers Grove, IL, USA). Liver damage and serum cytokines were determined by EnzyChrom Alanine Transaminase Assay (EALT-100; BioAssay Systems, Hayward, CA, USA) and enzyme-linked immunosorbent assays (ELISA) for mouse cytokines (Invitrogen, Carlsbad, CA, USA), respectively. For histology, paraffin-embedded sections (4 µm thick) stained by Hematoxylin and Eosin (H&E) from 10% formalin-fixed samples were evaluated. The scoring system of obesity-induced liver damage was used as the following; steatosis (0–3), lobular inflammation (0–3), and hepatocellular ballooning degeneration (0–2)^36^. The thickness of subcutaneous fat was determined following a previous publication^37^. For the detection of lipids in the liver, livers were sonicated (High-Intensity Ultrasonic Processor, Newtown, CT, USA) in 500 µl of ice-cold PBS containing protease inhibitor Cocktail (I3786) (Sigma-Aldrich) and measured lipids from the supernatant by the quantification assay for triglyceride and total cholesterol (Sigma-Aldrich).

In addition, oxidative stress in the liver was evaluated following a previous study^38^. Briefly, livers were homogenized in radioimmunoprecipitation assay (RIPA) with protease inhibitor Cocktail (I3786) (Sigma-Aldrich) on ice before measuring an oxidative stress molecule, malondialdehyde (MDA), (Cayman Chemical Company, Ann Arbor, MI, USA). For an anti-oxidant molecule, livers were sonicated in 2-(N-morpholino) ethanesulfonic acid (MES) buffer (Sigma-Aldrich) before the measurement of glutathione (GSH) (Cayman Chemical Company) from the supernatant. Furthermore, the mucin production from the colon was evaluated by real-time polymerase chain reaction (PCR). Briefly, the total RNA was prepared from the colon samples with an RNA-easy mini kit (Qiagen, Hilden, Germany), quantified by NanoDrop 100 Spectrophotometer (Thermo Fisher Scientific) before the determination of gene expression. Total RNA reverse transcription was processed with a High-Capacity cDNA Reverse Transcription (Thermo Fisher Scientific). Samples were performed using SYBR Green PCR Master Mix for quantitative real-time PCR with QuantStudio6 Flex Real-time PCR System (Thermo Fisher Scientific), respectively. The gene expression of Mucin-2 (*MUC2*) (forward 5′-CGACACCAGGGATTTCGCTTAAT-3′; reverse 5′-CACTTCCACCCTCCCGGCAAAC-3′) were determined in terms of relative quantitation of the comparative threshold (delta-delta Ct) method (2-ΔΔCt) as normalized by *β-actin* (an endogenous housekeeping gene) (forward 5′-CGGTTCCGATGCCCTGAGGCTCTT-3′; reverse 5′-CGTCACACTTCATGATGGAATTGA-3′).

### Mouse fecal analysis and the ex vivo experiment on feces

Fecal pH was evaluated using 1 g of feces thoroughly mixed with 2 ml of water before centrifugation at 4,000 rpm for 3 min. Then, the pH of the supernatant was measured by pH meter (Orion 4-star, Benchtop pH/Conductivity Thermo Fisher Scientific). Besides, the bacterial abundance of *Akkermansia muciniphila*, the beneficial bacteria against obesity^39^, and *Lactobacillus* spp. in several segments of mouse intestines with fecal contents were evaluated by PCR. Total DNA was extracted by a QIAamp fast DNA Stool Mini Kit (Qiagen, Hiden, Germany), as per the manufacturer’s instructions. Primers for the variable regions of the 16 s ribosomal RNA (rRNA) gene sequence of *A. muciniphila* bacteria^40^ were: AM1, 5′- CAGCACGTGAAGGTGGGGAC-3′; AM2, 5′-CCTTGCGGTTGGCTCAGAT-3′ and for *L.rhamnosus*^41^ were rham 5′-TGCATCTTGATTTAATTTTG-3′; Y2 5′-CCCACTGCTGCCTCCCGTAGGAGT-3′. The relative quantitation was normalized to the housekeeping 16S bacterial rRNA gene (forward 5′-ACGCAACTGACGAGTGTGAC-3′; reverse, 5′-GATCGCGACACCGAACTAAT-3′). The comparative cycle threshold against the gene expression of these bacteria was demonstrated.

In addition, to further determine the association between *Lactobacillus* spp. and *Akkermansia* spp., cecal feces from 8 wk-HFD-administered mice (30 mg) were anaerobically cultured under anaerobic conditions by gas generation sachets (AnaeroPack; Mitsubishi Gas Chemical, Japan) using Brain Heart infusion media (BHI; an enriched media for *Akkermansia* spp.) or De Man, Rogosa and Sharpe media (MRS; an enriched media for *Lactobacillus* spp.) (Oxoid Tryptone; Thermo Fisher Scientific) with or without LA5 for 48 h at 37 °C before detecting bacterial gene expression by PCR as previously mentioned.

### Fecal microbiome analysis

Feces from 9 mice (0.25 g per mouse) from different cages in each experimental group were divided into three samples per group (3 mice per sample) before performing microbiota analysis following a previous protocol^42^. In short, metagenomic DNA was extracted from 0.25 g feces by DNeasyPowerSoil Kit (Qiagen, MD, USA). The Universal prokaryotic 515F (forward; (5′-GTGCCAGCMGCCGCGGTAA-3′) and 806R (reverse; 5′-GGACTACHVGGGTWTCTAAT-3′), with appended Illumina adapter and Golaybarcode sequences, were used for 16S rRNA gene V4 library construction and sequenced using Miseq 300 platform (Illumina, San Diego, CA, USA) at Omics Sciences and Bioinformatics Center, and Microbiome Research Unit for Probiotics in Food and Cosmetics, Chulalongkorn University. Raw sequences were quality processed and operational taxonomic unit (OTU) classified following Mothur’sstandard operating platform procedures^43,44^. Bioinformatic analyses included good’s coverage, alpha diversity (e.g. Chao), and beta diversity (e.g. non-metric multidimensional scaling (NMDS)). Linear discriminant analysis Effect Size (LEfSe) and meta-stats were also performed to determine species marker and unique representing species of the interested group, respectively^43,45^. In addition, nucleic acid sequences in this study were deposited in an NCBI open-access sequence read archive database (SRA), accession number SRP286245.

### In vitro experiment

Because serum endotoxin could be detected in obesity^25^ and saturated fatty acid-activated hepatocytes, the conditioned media from LA5 were incubated in HepG2 hepatocyte cell-line with palmitic acid (a representative saturated fatty acid) and/or endotoxin to see an impact of LA5 on hepatocytes. For the preparation of LA5 conditioned-media, LA5 at 0.1 McFarland standards (OD600) were incubated anaerobically for 48 h before supernatant collection and filtered with a 0.22-µm membrane filter (Minisart; Sartorius, Göttingen, Germany). Next, 500 µl of the preparation was concentrated by speed vacuum drying at 40 °C for 3 h (Savant Instruments, NY) and the concentrated pellets were re-suspended with HyClone Dulbecco's Modified Eagle Medium (DMEM) high-glucose medium (HyClone; Laboratories, UT, USA). The small molecules of condition media were selected by the filtration with membrane filters (50 and 100 kDa) (Milipore; Merck, Darmstadt, Germany) and stored at 20 °C before use.

HepG2, a human hepatoma cell-line, (ATCC HB-8065; Thermo Fisher Scientific), was maintained in DMEM with 10% fetal bovine serum (FBS), 1% penicillin/streptomycin antibiotics, and 1% Sodium pyruvate in a humidified atmosphere of 5% CO_2_ at 37 °C. HepG2 at 1 × 10^4^ cells/ml, 0.5 mM of palmitic acid (PA) (Sigma-Aldrich), and 1 µg/ml of lipopolysaccharide (LPS from *E. coli* O55:B5) (Sigma-Aldrich) with or without LA5 conditioned media in 96-well plate with DMEM at 37 °C before supernatant and cell collection. Supernatant cytokines were then determined using ELISA for human cytokines (TNF-α, IL-8, and IL-10) (R&D System, Minneapolis, MN, USA) and intracellular lipid content was determined by 0.3% Oil Red O staining (Sigma-Aldrich) with the evaluation by ImageJ (National Institute of Health, Bethesda, MD, USA) in 10 randomized fields^46^.

### Statistical analysis

Mean ± standard error (SE) was used for data presentation. The differences between groups were examined for statistical significance by one-way analysis of variance (ANOVA) followed by Tukey’s analysis or Student’s *t-*test for comparisons of multiple groups or 2 groups, respectively. All statistical analyses were performed with SPSS 11.5 software (SPSS, IL, USA) and Graph Pad Prism version 7.0 software (La Jolla, CA, USA). A *p*-value of < 0.05 was considered statistically significant.

## Results

### *Lactobacillus acidophilus* LA5 attenuated obesity in high-fat diet mice

**Figure 1:**
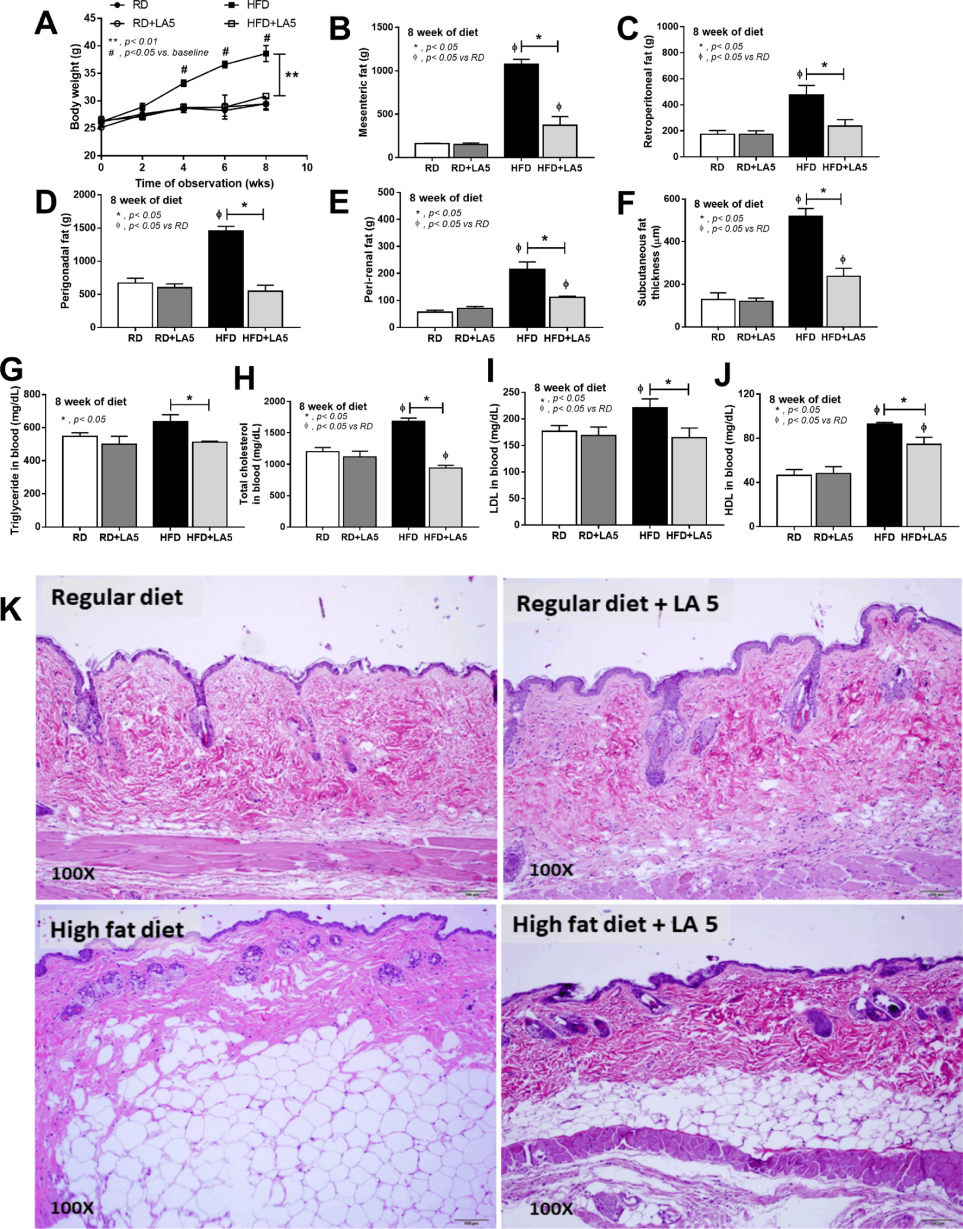
Characteristics of mice fed with regular diet (RD) or high-fat diet (HFD) with or without *Lactobacillus acidophilus* LA5 (LA5) as determined by body weight (**A**), adipose tissue depots in several sites (**B**–**E**), subcutaneous fat thickness (**F**), fasting blood lipid profiles (**G**–**J**) and the representative figures of the subcutaneous fat thickness (original magnification × 200) (**K**) were demonstrated (n = 6–8/group for **A**–**J**). LDL, low-density lipoprotein; HDL, high-density lipoprotein.

**Figure 2:**
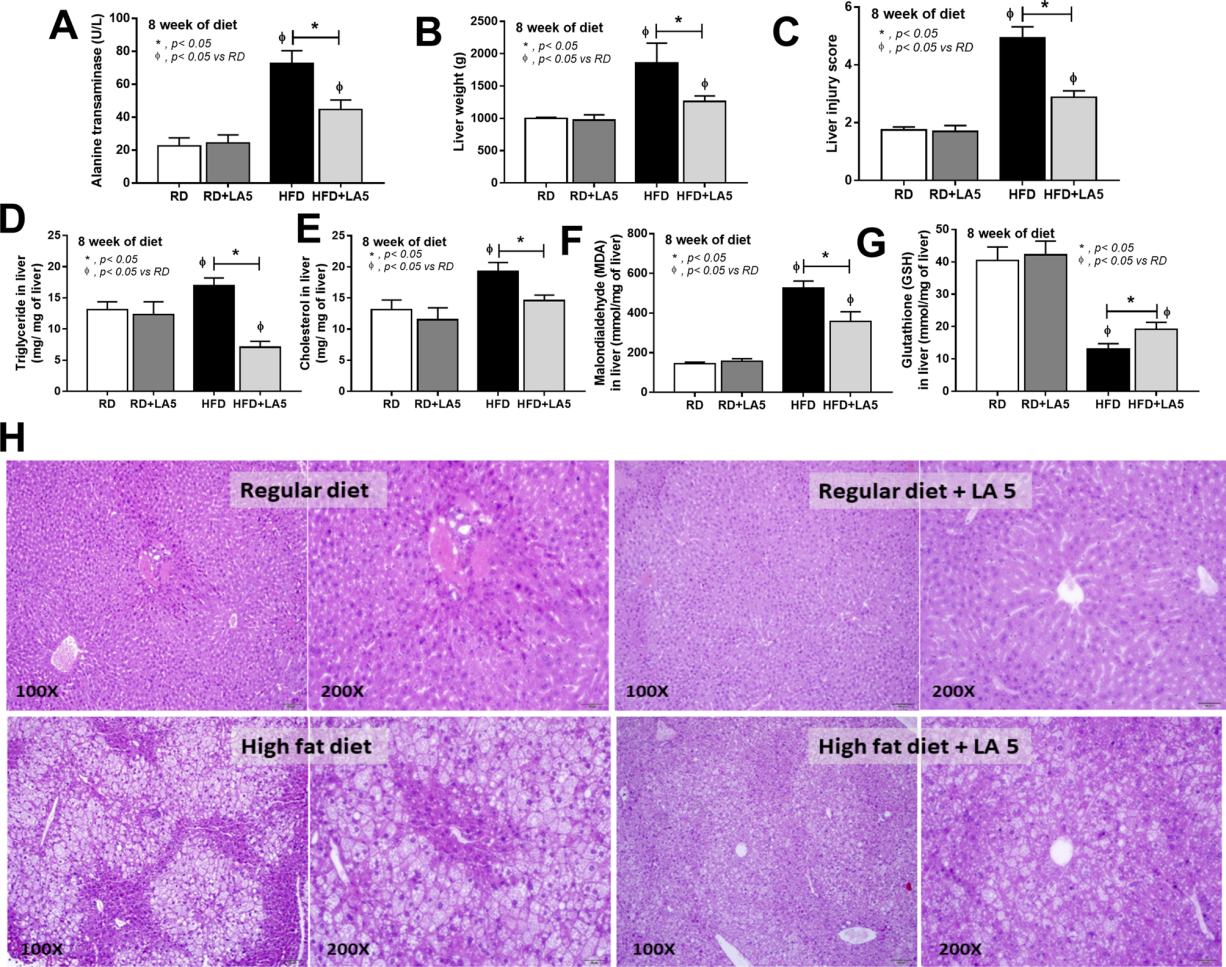
Characteristics of liver injury in mice fed with regular diet (RD) or high-fat diet (HFD) with or without *Lactobacillus acidophilus* LA5 (LA5) as determined by alanine transaminase (ALT) (**A**), liver weight (**B**), histological liver injury score (**C**), lipid components in the liver including triglyceride, total cholesterol (**D**,**E**), oxidative stress (malondialdehyde; MDA) in the liver (**F**), an anti-oxidant molecule (glutathione) in the liver (**G**) and the representative pictures of liver histology (**H**) were demonstrated (n = 6–8/group for **A**–**G**). Severe lipid accumulation (steatohepatitis) was indicated in HFD mice.

**Figure 3:**
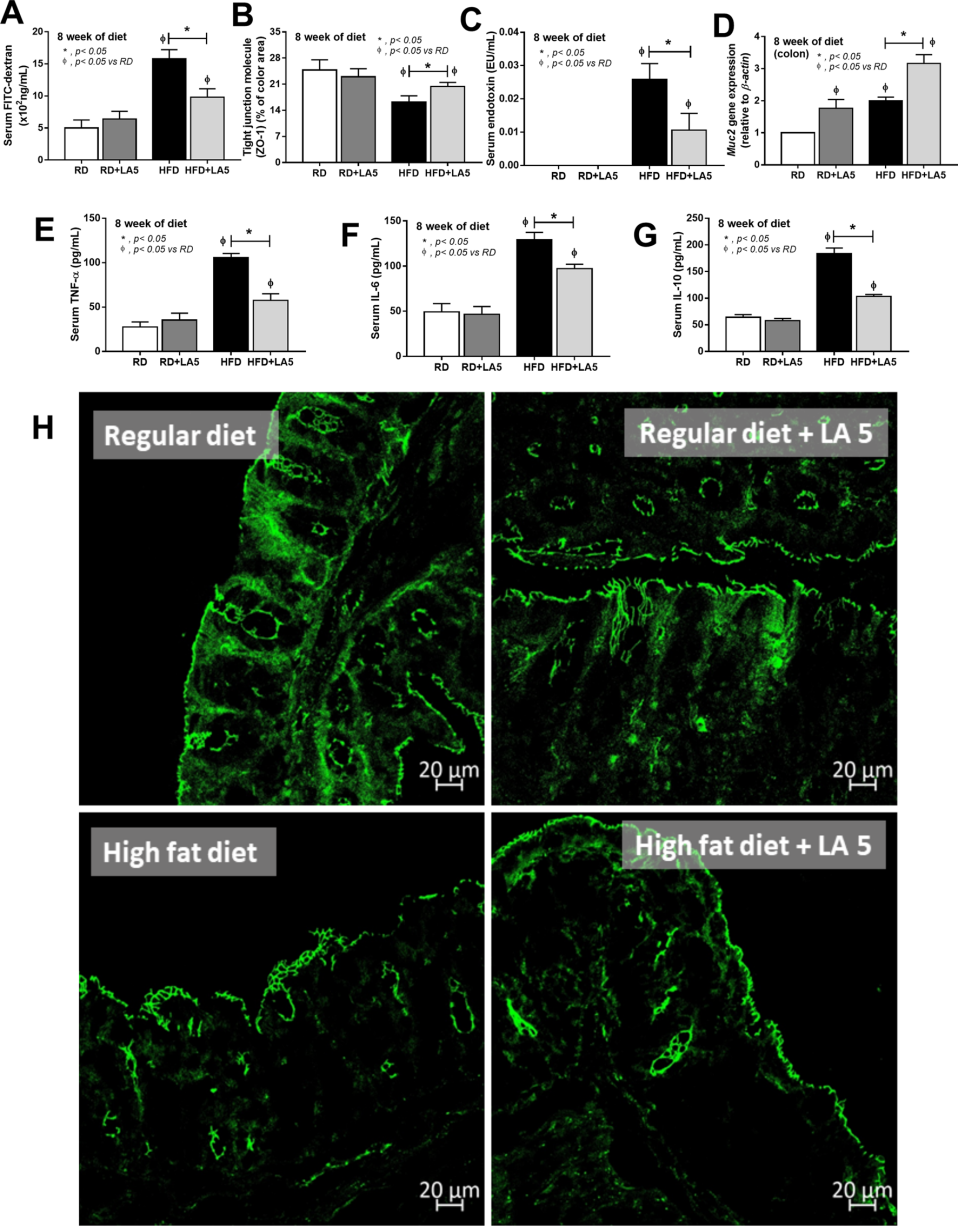
Characteristics of gut leakage and systemic inflammation in mice fed with regular diet (RD) or high-fat diet (HFD) with or without *Lactobacillus acidophilus* LA5 (LA5) as determined by FITC-dextran assay (**A**), a tight junction protein (zonaoclludens-1; ZO-1) on intestinal mucosa (**B**), serum endotoxin (**C**), gene expression of mucin-2 (*muc2*) in the colon (**D**), serum cytokines (**E**–**G**) and the representative fluorescent pictures of ZO-1 staining in cecal mucosa (**H**) were demonstrated (n = 6–8/group for **A**–**G**).

*Lactobacillus acidophilus* LA5 (LA5) attenuated obesity in mice as determined by bodyweight and visceral fat deposition in several sites including mesentery, retro-peritonium, peri-gonadal, peri-renal and subcutaneous fat along with lipid profiles (TG, total cholesterol, HDL and LDL) (Fig. 1A–K) supported several studies that utilizing LA5-combined probiotics^26,27^. In addition, LA5 also attenuated obesity-induced liver injury as evaluated by liver enzyme, liver weight, the histological score of steatohepatitis, the lipid composition of livers, and the oxidative stress (Fig. 2A–H). Moreover, LA5 improved gut permeability defect (gut leakage) and leaky gut-induced systemic inflammation as indicated by FITC-dextran assay, ZO-1tight junction protein, endotoxemia, colon mucin production (gene expression of *muc2*) and serum cytokines (TNF-α, IL-6, and IL-10) (Fig. 3A–H). Because (i) Gram-negative bacteria in the gut is a source of endotoxin (LPS)^7^, (ii) obesity enhances endotoxemia from gut translocation^25^ and (iii) molecules from gut translocation is possibly directly transported to the liver through portal veins^7^, the reduced serum endotoxin in LA5 administered-obese mice implies the improved gut dysbiosis.

### *Lactobacillus acidophilus *LA5 attenuated gut dysbiosis and promoted beneficial *Akkermansia* spp. in high-fat diet mice

**Figure 4:**
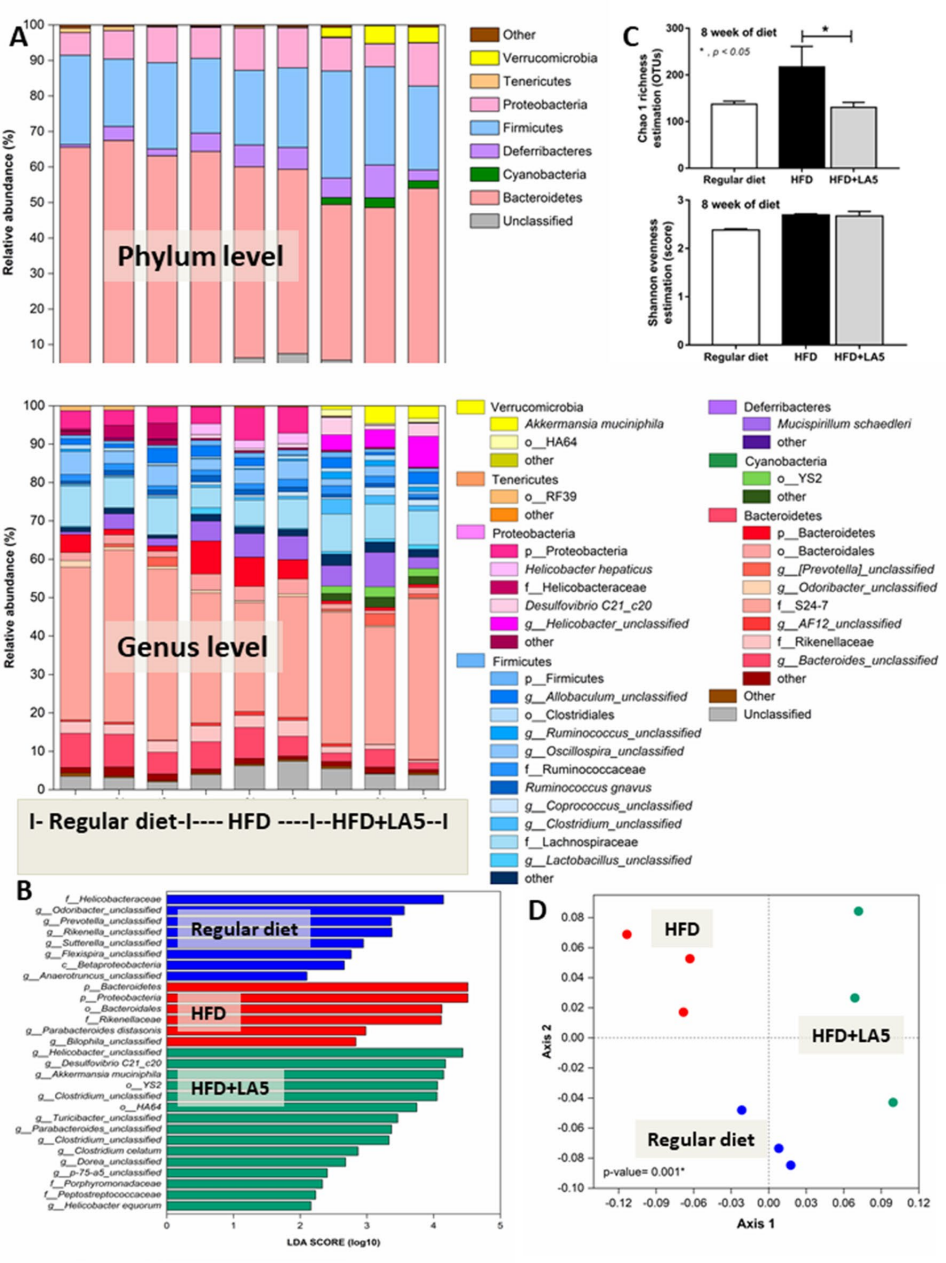
Gut microbiota analysis from feces of mice fed with a regular diet, high-fat diet (HFD), and HFD with *Lactobacillus acidophilus* LA5 (LA5) as determined by the relative abundance of bacterial diversity at phylum and genus (**A**), the possibly unique bacteria in each group by Linear discriminant Effect Size (LEfSe) analysis (**B**), the alpha diversity by Chao 1 richness estimation and Shannon evenness analysis (**C**) and non-metric multidimensional scaling (NMDS) of bacteria based on the species taxonomic level (**D**).

**Figure 5:**
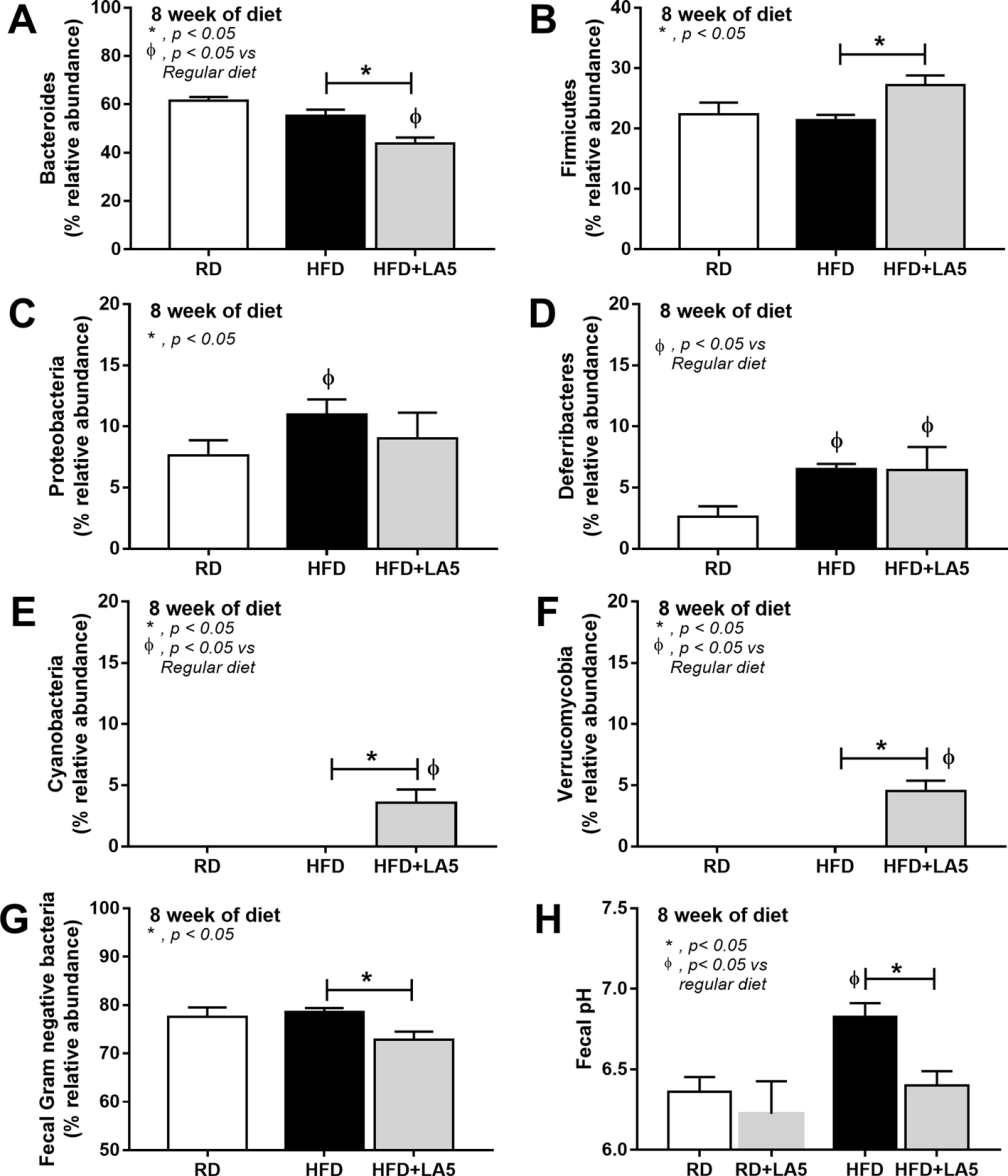
Gut microbiota and fecal analysis from feces of mice fed with a regular diet, high-fat diet (HFD), and HFD with *Lactobacillus acidophilus* LA5 (LA5) as determined by the relative abundance of bacterial diversity at phylum with bar graph presentation (**A**–**F**), total Gram-negative bacterial abundance in feces (**G**) and fecal pH (**H**) were demonstrated.

From fecal microbiome analysis, HFD increased Proteobacteria, the pathogenic Gram-negative bacteria, without causing a difference in Bacteroides, the most abundance Gram-negative bacteria in feces, nor the total Gram-negative bacteria in feces when compared with the regular diet group (Figs. 4A, 5A–G). However, at a genus level, bacteria in the Bacteroidales group such as S24-7, AF-12, and *Prevotella* spp. were predominant in HFD mice compared with the regular diet group (Fig. 4A lower and Table 1), supporting obesity-enhanced Bacteroides bacteria as previously reported^47^. Interestingly, HFD induced endotoxemia (Fig. 1J), despite the similar Gram-negative bacterial burdens in feces (fecal LPS burdens) compared with regular diet mice (Fig. 5G), was the consequence of gut mucosal injury by pathogenic bacteria such as *Desulfovibrio*spp. and RF32^48,49^ in Proteobacteria group (Table 1). This implies a difference between fecal microbiota in the genus level of these groups. The microbiota analysis among regular diet, HFD, and HFD + LA5 groups are shown in Tables 1, 2, and 3.

**Table 1:** Comparison of fecal microbiota in genus level between regular diet vs high fat diet (HFD) mice.

Bacteria	Relative abundance (%)		p-value*	Direction^#^
	Regular diet	HFD		
g__Bilophila_unclassified	0.000	0.135	0.002	Up
Helicobacter hepaticus	0.003	2.547	0.003	Up
p__Firmicutes	0.814	1.351	0.004	Up
o__Bacteroidales	2.009	3.927	0.005	Up
g__Clostridium_unclassified	0.001	0.158	0.007	Up
f__Porphyromonadaceae	0.000	0.014	0.009	Up
Desulfovibrio C21_c20	0.059	0.855	0.012	Up
Parabacteroides distasonis	0.000	0.184	0.023	Up
Mucispirillum schaedleri	2.376	5.842	0.025	Up
g__AF12_unclassified	0.411	0.882	0.026	Up
o__RF32	0.004	0.043	0.030	Up
g__Coprococcus_unclassified	0.253	0.812	0.038	Up
p__Bacteroidetes	2.276	6.975	0.039	Up
unclassified	2.830	5.977	0.043	Up
f__Lactobacillaceae	0.016	0.051	0.047	Up
f__Rikenellaceae	2.835	3.789	0.048	Up
g__Prevotella_unclassified	0.530	0.063	0.001	Down
f__S24-7	43.375	31.063	0.008	Down
c__Betaproteobacteria	0.095	0.017	0.029	Down

*p < 0.05 Regular diet vs HFD.

^#^Direction of change in HFD compared with regular diet.

**Table 2:** Comparison of fecal microbiota in genus level between high fat diet (HFD) vs probiotics-treated HFD (HFD + LA5) mice.

Bacteria	Relative abundance (%)		p-value*	Direction^#^
	HFD	HFD + LA5		
o__YS2	0.015	2.226	0.002	Up
o__Desulfovibrionales	0.011	0.273	0.005	Up
f__Peptostreptococcaceae	0.001	0.031	0.008	Up
g__Dorea_unclassified	0.015	0.106	0.012	Up
f__Porphyromonadaceae	0.014	0.036	0.014	Up
g__Helicobacter_unclassified	0.081	5.448	0.019	Up
f__Lachnospiraceae	6.560	9.502	0.028	Up
g__p-75-a5_unclassified	0.000	0.051	0.041	Up
g__Prevotella_unclassified	0.063	0.179	0.043	Up
g__Parabacteroides_unclassified	0.005	0.470	0.049	Up
Helicobacter hepaticus	2.547	0.005	0.001	Down
o__Bacteroidales	3.927	1.325	0.003	Down
p__Bacteroidetes	6.975	0.826	0.006	Down
p__Proteobacteria	6.687	0.084	0.009	Down
f__Rikenellaceae	3.789	1.237	0.010	Down
g__Ruminococcus_unclassified	0.001	0.000	0.016	Down
g__Odoribacter_unclassified	0.609	0.298	0.023	Down
c__Gammaproteobacteria	0.020	0.004	0.024	Down
[Ruminococcus] gnavus	1.417	0.794	0.030	Down
Parabacteroides distasonis	0.184	0.002	0.031	Down
g__Alistipes_unclassified	0.161	0.037	0.038	Down
g__Bacteroides_unclassified	6.720	2.861	0.045	Down
Proteus myxofaciens	0.002	0.000	0.047	Down

*p < 0.05 HFD vs HFD + LA5.

^#^Direction of change in HFD + LA5 compared with HFD.

**Table 3:** Comparison of fecal microbiota in genus level between regular diet vs probiotics-treated HFD (HFD + LA5) mice.

Bacteria	Relative abundance (%)		p-value*	Direction^#^
	Regular diet	HFD + LA5		
g__Clostridium_unclassified	0.427	2.771	0.003	Up
o__YS2	0.010	2.226	0.004	Up
o__Desulfovibrionales	0.006	0.273	0.009	Up
f__Peptostreptococcaceae	0.000	0.031	0.010	Up
g__Lactobacillus_unclassified	0.194	0.893	0.015	Up
g__Ruminococcus_unclassified	0.220	1.543	0.018	Up
g__Helicobacter_unclassified	0.141	5.448	0.019	Up
Lactobacillus reuteri	0.000	0.136	0.023	Up
Alistipes finegoldii	0.002	0.015	0.034	Up
Desulfovibrio C21_c20	0.059	3.329	0.038	Up
g__Parabacteroides_unclassified	0.002	0.470	0.043	Up
g__Clostridium_unclassified	0.001	0.442	0.045	Up
p__Proteobacteria	4.243	0.084	0.001	Down
g__Sutterella_unclassified	0.196	0.028	0.002	Down
g__Prevotella_unclassified	0.530	0.179	0.007	Down
Proteus myxofaciens	0.010	0.000	0.008	Down
o__Burkholderiales	0.006	0.000	0.013	Down
g__Flexispira_unclassified	0.105	0.000	0.020	Down
c__Betaproteobacteria	0.095	0.004	0.021	Down
g__Alistipes_unclassified	0.123	0.037	0.022	Down
c__Gammaproteobacteria	0.005	0.004	0.023	Down
g__Bacteroides_unclassified	7.581	2.861	0.029	Down
g__Oscillospira_unclassified	4.676	1.767	0.034	Down
g__Desulfovibrio_unclassified	0.296	0.004	0.036	Down
Butyricicoccus pullicaecorum	0.148	0.070	0.044	Down

*p < 0.05 Regular diet vs HFD + LA5.

^#^Direction of change in HFD + LA5 compared with Regular diet.

In LA5 administration with HFD, there was an increase in Firmicutes (beneficial Gram-positive anaerobes), Verrucomycobia (Gram-positive anaerobes with some benefits) and Cyanobacteria (bacteria that has not been possible to isolate)^50–52^ with a decrease in Bacteroides and total Gram-negative bacterial burdens in feces (Figs. 4A and 5A–G). The reduced fecal burdens of total Gram-negative bacteria (Fig. 5G) may be partly responsible for less severe endotoxemia in LA5-treated HFD mice (Fig. 1J). Interestingly, LA5 administration reduced Gammaproteobacteria, a group of pathogenic bacteria, including *Pseudomonas* spp., *Klebsiella* spp., and *Escherichia* coli, which also might be beneficial (Tables 2, 3). Additionally, the Linear discriminant analysis Effect Size (LEfSe) demonstrates the variation in bacteria among groups including (i) *Helicobacter*, *Odoribacter,* and *Prevotella* in the regular diet group, (ii) Bacteroides and Proteobacteria in HFD, and iii) *Helicobacter*, *Desulfovibrio*, *Akkermansia,* and *Clostridium* in HFD with LA5 (Fig. 4B). Moreover, HFD increased bacterial diversity as determined by Chao-1 richness estimation (variety of species) with similar evenness estimation (proportions of the individual species) in comparison to regular diet mice (Fig. 4C). Furthermore, the difference in fecal microbiome was demonstrated by a distinct separation in non-metric multidimensional scaling (NMDS) of bacteria based on the species taxonomic level (Fig. 4D). Notably, HFD increased fecal pH possibly due to reduced short-chain fatty acids (SCFAs)-induced bacteria^53^ but the acidification property of LA5 restored fecal pH in HFD-administered mice (Fig. 5H).

**Figure 6:**
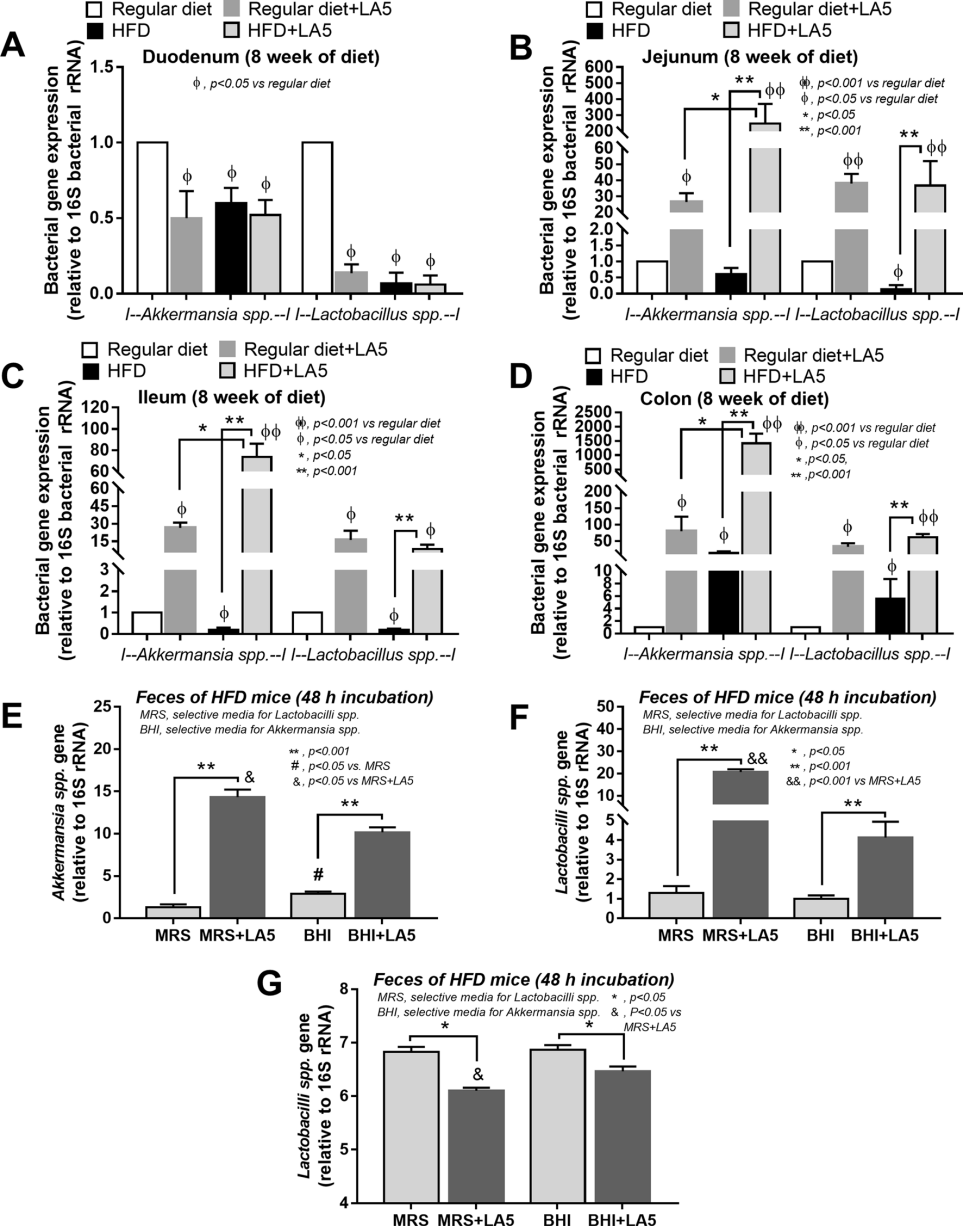
The abundance of bacterial gene expression in several parts of mouse intestines with fecal contents from mice fed with a regular diet, high-fat diet (HFD), and regular diet or HFD with *Lactobacillus acidophilus* LA5 (LA5) as determined by polymerase chain reaction (PCR) were demonstrated (**A–D**) (n = 5–6/group). Additionally, the abundance of bacterial gene expression in feces from HFD mice with or without LA5 after 48 h incubation in De Man, Rogosa, and Sharpe media (MRS; an enriched media for *Lactobacilli*) or Brain Heart Infusion media (BHI; an enriched media for *Akkermansia*) by PCR (**E**,**F**) and the pH of these samples (**G**) were demonstrated (independent triplicate experiments were performed).

Among all of these bacteria enhanced by LA5 administration, *Akkermansia muciniphila* is known to attenuate obesity^39^. Hence, we analyzed the presence of *A. muciniphila* and *L. Acidophilus* in the feces from various sections of the intestine by PCR. Without LA5, HFD mildly increased both bacteria only in the colon at approximately five–tenfold of the regular diet group (Fig. 6A–D). On the other hand, both bacteria were profoundly elevated in the jejunum, ileum, and colon, but not the duodenum in a regular diet or HDF with LA5 administration (Fig. 6A–D) implying the promotion of *A. muciniphila* by LA5. Notably, *A. muciniphila* in non-obese regular diet mice with LA5 was lower than HFD with LA5 (Fig. 6A–D) suggesting an influence of obesity with LA5 on *A. muciniphila*. Furthermore, the ex vivo incubation of feces from cecum with or without LA5 demonstrated the increased *Akkermansia* spp. as detected by PCR with LA5 incubation in either the enriched media for *Lactobacilli* (MRS) or the media for *Akkermansia* (BHI) (Fig. 6E,F). After incubation, the pH of the samples with LA5 demonstrated a lower pH than non-LA5 samples (Fig. 6G) implying a possible pH-associated promotion of *Akkermansia* growth (maximized growth at pH 6.5)^54^. Accordingly, the abundance of *Akkermansia* in the samples with LA5 co-incubation in MRS, an enriched media for *Lactobacilli*, was even higher than the abundance in BHI, an *Akkermansia*-enriched media, without LA5 (Fig. 6E,F).

### *Lactobacillus acidophilus* LA5 attenuates fatty acid-induced hepatocyte inflammation

**Figure 7:**
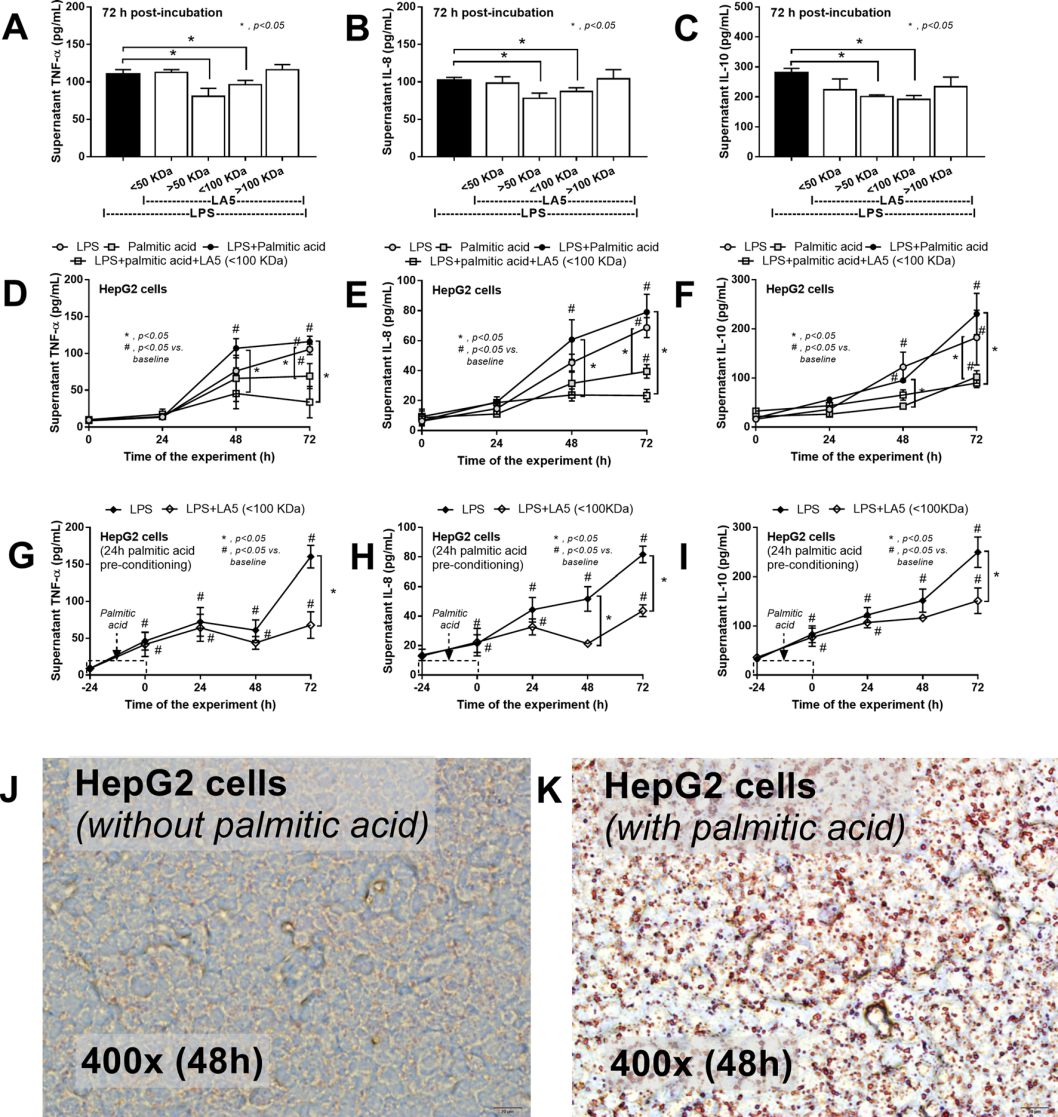
Supernatant cytokines (TNF-α, IL-8, and IL-10) from HepG2 cells (hepatocytes) after activation by lipopolysaccharide (LPS) with condition media of *Lactobacillus acidophilus* LA5 (LA5) after filtered with membrane filters at 50 and 100 kDa (**A**–**C**), byLPS and palmitic acid (PA; a representative saturated fatty acid), alone or in combination, with or without LA5 condition media after filtered with 100 kDa membrane filters (**D–F**) were demonstrated. Besides, the supernatant cytokines from LPS-activated HepG2 cells after 24 h PA pre-conditioning with or without filtrated LA5 condition media (100 kDa) (**G**–**I**) and the representative pictures of HepG2 cell without or with PA incubation (**J**,**K**) were indicated (independent triplicate experiments were performed).

Because direct activation of LPS and *Lactobacilli*-producing molecules on hepatocytes during obesity-induced gut translocation through portal vein is possible^7,55^, in vitro LPS activation on hepatocytes with or without *Lactobacillus* condition media was performed. Indeed, *Lactobacillus* condition media with molecular weight > 50 and < 100 kDa—but not < 50 and > 100 kDa—reduced cytokine production from HepG2 cells at 72 h post-incubation (Fig. 7A–C). Thus, *Lactobacillus* condition media with molecular weight < 100 kDa was used in further experiments. On the other hand, palmitic acid (PA) induced lipid accumulation with mild supernatant cytokine production in comparison to LPS production, in HepG2 cells at 72 h post-incubation (Fig. 7D–F). The incubation of PA together withLPS (LPS + PA) induced similar cytokine responses to LPS activation alone and the LA5 supernatant was able to reduce the level of these cytokines at 48 and 72 h post-incubation (Fig. 7D–F). In parallel, the pre-incubation of PA for 24 h before LPS activation in HepG2 cells also enhanced supernatant cytokines and was attenuated by supernatant of LA5 (Fig. 7G–K) suggesting that LA5 produces anti-inflammatory substances.

## Discussion

The administration of *Lactobacillus acidophilus* LA5 (LA5) together with a high-fat diet (HFD) attenuated hyperlipidemia, steatohepatitis, and obesity in a mouse model through the improved gut dysbiosis and the production of anti-inflammatory substances. Although only male mice were used for the experiments, these data were a proof of concept demonstrating the LA5 anti-obesity property.

### Dysbiosis and metabolic endotoxemia in obese mice

The characteristics of obesity in HFD-administered mice were overweight, increased fat accumulation, hyperlipidemia, liver injury (liver weight, steatohepatitis, and elevated liver enzymes), and gut permeability defect (FITC-dextran assay, reduced tight junction protein, and increased serum LPS). Obesity-induced liver injury is partly stimulated by a direct LPS activation on hepatocytes as a result of gut permeability defects^7^. Because inflammatory responses against pathogens (eg. LPS) are much stronger than the responses toward self-antigens^6^, obesity-induced endotoxemia might be a fundamental activator that leads to major complications^56^. Indeed, HFD enhances fecal basification (increased fecal pH) possibly through amplified bile production and reduction of short-chain fatty acids (SCFAs)^57^, which increase Proteobacteria (mucosal invasive Gram-negative organisms^58,59^) and enhance Deferribacteres (rodent gut-pathogens^60^) but not Bacteroidetes (Gram-negative anaerobes in pathogenic conditions^61^) nor fecal burdens of total Gram-negative bacteria. This data supports that HFD enhances endotoxemia^62^ through dysbiosis-induced intestinal mucosal injury^63,64^, but not increases LPS burdens in the gut, and the endotoxemia enhances liver injury and promotes systemic inflammation. Hence, the attenuation of dysbiosis and/ or gut-leakage may be a direct adjunctive treatment against obesity-induced inflammation and other complications.

### *Lactobacillus acidophilus* LA5 attenuated obesity and gut dysbiosis

Probiotics are the most common treatment for gut dysbiosis^22,24,25,50^, attenuating obesity through several mechanisms such as the induction of energy-efficient microbiota, production of SCFAs, and promotion of intestinal hormones with anti-obesity properties^65^. Among several probiotics, *Lactobacillus acidophilus* demonstrates robust lactic acid production in comparison to other strains^66^ that may strongly alter HFD-induced dysbiosis. The non-difference in *Lachnospiraceae* bacteria (*Lactobacillus* group) in fecal microbiome analysis (Fig. 4A) between mice with versus without LA5 administration demonstrated the intestinal attachment property of a good probiotics^67^. Indeed, LA5 was able to normalize fecal pH, decrease Bacteroidetes, a predominant group of bacteria in pathogenic conditions^61^, reduce total Gram-negative bacterial strain in feces as well as attenuate endotoxemia. Additionally, LA5 enhanced Firmicutes (Gram-positive bacteria that prominently identified in healthy gut^68^) and Verrucomycobia (a phylum of bacteria that inversely correlated to several intestinal abnormalities^54^) in feces that possibly attenuated dysbiosis and improved intestinal mucosal integrity. Interestingly, *Akkermansia muciniphila* is a bacterium of the phylum Verrucomicrobia that demonstrates several advantages including anti-obesity properties^30,69^. While the intestinal mucus layer in obese mice is reduced by HFD^70^, *A. muciniphila* increases the intestinal mucin thickness and attenuates gut-leakage^71^, despite having a mucin-degrading function^40^. When LA5 was administered, *A. muciniphila* in the colon increased by 2,000 folds (as determined by bacterial nucleic acid) in comparison to the control group, alluding to another mechanism of gut-leakage attenuation.

Interestingly, the optimal pH for *Akkermansia* growth is 6.5^54^ but the pH of HFD feces was higher than 6.5 (Fig. 5H) possibly due to the reduction of short-chain fatty acids^53^. Indeed, an abundance of *Akkermansia* spp. in fecal samples with LA5 using MRS media (the *Lactobacilli* enriched media) was higher than the culture in BHI (the *Akkermansia* enriched media) (Fig. 6E–G) supporting an impact of pH on *Akkermansia* growth. Although *Lactobacilli* might produce several promoting factors for *Akkermansia* growth, the acidification property of *Lactobacilli* might be one of these factors. On the other hand, in non-obese regular diet mice, LA5 also increased *A. muciniphila* (without fecal acidification) and induced colon mucin production (*muc2* gene expression) supported LA5 benefit on the enhanced-intestinal mucosal integrity through *A. muciniphila* induction in the healthy host. Meanwhile, the lower *A. muciniphila* in LA5-administered non-obese mice than the LA5-administered obese mice, despite the none *A. muciniphila* in HFD mice without LA5, implied the different mechanisms of LA5-enhanced *A. muciniphila* between obese mice versus non-obese mice possibly through fecal acidification versus some fat derivatives, respectively. Hence, the beneficial effects of probiotics in healthy and unhealthy mice might be different. Unfortunately, the fecal microbiome analysis in feces from LA5-administered regular diet mice (non-obese mice) was not performed here. Nevertheless, the LA5 administration in obesity might be an interesting growth promotion of the beneficial bacteria that are difficult to cultivate including *A. muciniphila*^30^*.* More studies are interesting.

### *Lactobacillus acidophilus* LA5 attenuated hepatic injury through the production of anti-inflammatory molecules

Although molecules with a molecular weight (MW) larger than 600 Da are kept inside the gut by intestinal integrity^7^, gut leakage allows these larger molecules to translocate through the gut. During gut leakage, beneficial molecules may be translocated alongside harmful molecules. For instance, LA5 produces anti-inflammatory molecules with MW of 50–100 kDa—similar to the MW of LPS^7^—that decreases supernatant cytokines of LPS-activated HepG2 cells via co-incubation or post-incubation with palmitic acid, a representative saturated fatty acid. Thus, hepatocytes may be activated by both LPS and anti-inflammatory molecules from LA5 from gut leakage. Further studies into the role of these molecules in reducing hepatocyte injury may yield fascinating results.

**Figure 8:**
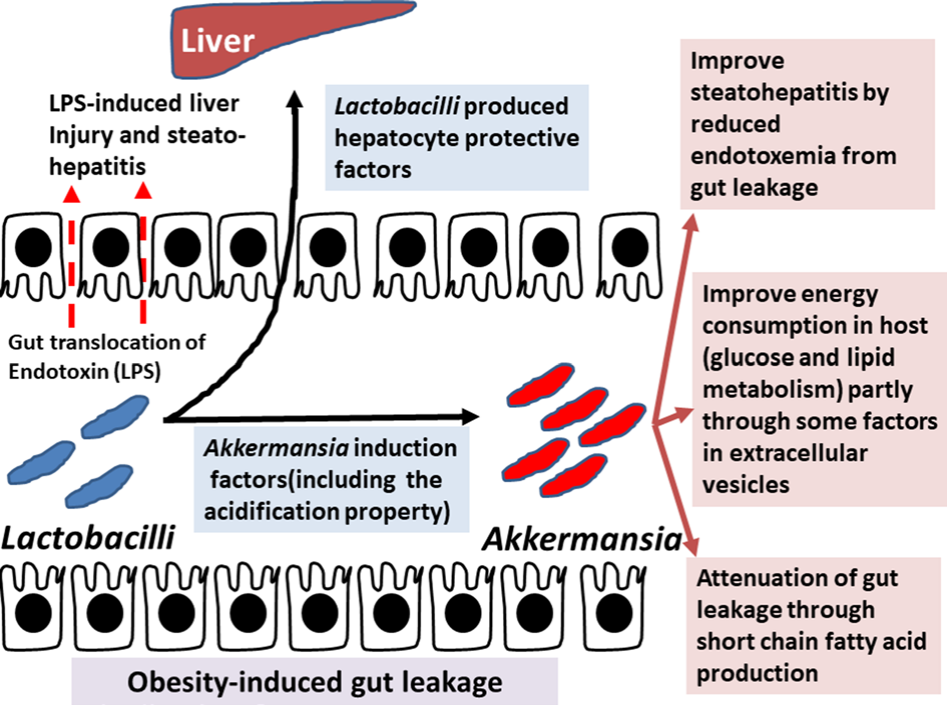
The proposed hypothesis demonstrates anti-obesity mechanisms of *Lactobacillus acidophilus* LA5 (LA5) directly through (i) promotion of *Akkermansia* spp. through an adjustment of fecal pH and (ii) attenuation of hepatic injury by gut translocation of the protective molecules. Besides, the known beneficial effects on obesity of LA5-promoted *Akkermansia* spp. including improved energy metabolism in the host, strengthened gut-permeability through short-chain fatty acids, and reduced hepatic injury by the attenuation of obesity-induced endotoxemia^39^ are the indirect anti-obesity effects of LA5.

In conclusion, HFD induced gut dysbiosis, gut leakage resulted in endotoxemia and systemic inflammation (Fig. 8). Then LA5 administration directly adjusted gut microbiome, in part, by acidifying fecal pH that promoted *Akkermansia* spp*.* and directly attenuated hepatic injury through *Lactobacilli* producing anti-inflammatory molecules. Additionally, promoted *Akkermansia* spp. is an indirect anti-obesity effect of LA5 through the known-mechanisms including improved energy consumption, produced SCFAs, gut leakage attenuation, reduced endotoxemia, and decreased liver injury^39^. Hence, the enhanced beneficial bacteria in the gut by probiotics is another interesting objective for probiotic utilization.
